# Sensory Recovery with Innervated and Noninnervated Flaps after Total Lower Lip Reconstruction: A Comparative Study

**DOI:** 10.1155/2013/643061

**Published:** 2013-12-05

**Authors:** Meltem Ayhan Oral, Kamuran Zeynep Sevim, Metin Görgü, Hasan Yücel Öztan

**Affiliations:** ^1^İzmir Katip Celebi University, Ataturk Research and Training Hospital, Department of Plastic and Reconstructive Surgery, 35360 Izmir, Turkey; ^2^Sisli Hamidiye Etfal Research and Training Hospital, Department of Plastic and Reconstructive Surgery, 34371 Istanbul, Turkey; ^3^Abant İzzet Baysal University, Department of Plastic and Reconstructive Surgery, 14280 Bolu, Turkey; ^4^Private Practice, Plastic Surgeon, 35590 Izmir, Turkey

## Abstract

This study compares sensory recovery after total lower lip reconstruction in a wide variety of flaps including bilateral depressor anguli oris flap, submental island flap, bilateral fan flaps, radial forearm flap, and pectoralis major myocutaneous flaps in a large number of patients. Spontaneous return of flap sensation was documented by clinical testing in the majority (3%) of patients who underwent total lower lip reconstruction. Sensory recovery occurred more often in patients with fasciocutaneous free flaps than in those with musculocutaneous flaps. Flap sensation to touch, two-point discrimination, and temperature perception was correlated with age, smoking, and radiation treated patients. We conclude that reasonable sensory recovery may be expected in noninnervated flaps, provided that the major regional sensorial nerve has not been sacrificed, and also provided that the patients age is relatively young and that enough surface contact area of the recipient bed is present without marked scarring. This trial was regestered with Chinese Clinical Trial Registry (Chi CTR) with ChiCTR-ONC-13003656.

## 1. Introduction

The reconstruction of an extensive lower defect is a difficult surgical challenge since both aesthetics and function of the lower third of the face have to be restored. The lip is a complex anatomical structure which includes a muscular layer which is a part of the oral sphincter, lying between a muscular layer and the overlying skin. In order to restore the three layers, several reconstructive procedures have been described, including local flaps from the cheeks, pedicled flaps from the chin, expanded cervical or jugal flaps, and finally fasciocutaneous free flap transfers [1–4]. Nevertheless, none of them provide an ideal solution regarding the appearance and function.

In this study we present 125 innervated and noninnervated flaps performed in bothclinics in 65 patients with stage 3 and further squamous cell carcinoma of the total lower lip. To date, 80 patients have been followed-up for a minimum of 1 year after the tumor resection and reconstruction with bilateral fan flaps, Karapandzic flaps, Nakajima flaps, Fujimori gate flaps, submental island flaps, bilateral depressor anguli oris myocutaneous flaps, pectoralis major myocutaneous flaps, and noninnervated radial forearm flaps was performed.

The comparative clinical recovery of sensation in these flaps and its relationship to articulation and perioral continence form the basis for this report.

## 2. Materials and Methods

From January 1, 1999, to August 2010, 80 patients, ranging in age from 7 to 82 years, underwent resection of stage 3 or greater squamous cell carcinoma of the lower lip.

The extent of resection of primary lesions included total lower lip and adjacent oral mucosa. Eighteen patients received postoperative irradiation and 50 patients received no further treatment.

Defects of the lower lip were reconstructed in 16 patients with 30 bilateral fan flaps, in 20 patients with 40 karapandzic flaps, in 5 patients with 10 Nakajima flaps, in 2 patients with 4 Fujimori Gate flaps, in 15 patients with 16 bilateral depressor anguli oris flaps, in 5 patients with submental island flaps, in 14 patients with radial forearm free flaps, and in 3 patients with pectoralis major musculocutaneous flaps.

The selection of the type of flap to be used for reconstruction in a particular patient was based on multiple factors, including size of the defect, the need and lack of bulk, the results of the Allen's test in the nondominant arm of the patients, the patient's preference after preoperative counseling, and preference of the surgeon. In this prospective study, no attempt was made in any patient to anastomose a neural stump in the recipient site to a nerve or nerves in the flap. Fifty of the patients were treated with surgery alone and 18 patients needed subsequent radiation therapy (70 Gy at the primary tumor site) (Table 3).

Surgical resection was combined with neck lymph-node dissection in 27 cases taking into consideration the patient's general health conditions.

All flaps selected for this study were total/near total lower lip defects in an attempt to minimize the confusion that might result from rapid ingrowth of surrounding nerves in smaller flaps.

The lingual and hypoglossal nerves were preserved carefully during neck dissection. Mean operating time was 2.5 hours.

All patients underwent sensory testing by the same examiner at least 3 months after surgery. Sensory function was assessed in the center of the flaps and on the contralateral side cheek as a control group. The tests were performed with the patient blinded. Patients were asked to acknowledge sensation by holding up their fingers depending on the sensation tested.

Superficial touch was tested by touching the flap with a cotton swab, hot and cold temperature discrimination was tested with immersing 2 test tubes in hot and cold water 5 minutes in hot water (≤40°C), 5 minutes in cold water (≥15°C), and two point discrimination was tested with Semmes-weinstein monofilaments.

The following factors and their relationship with flap sensory recovery were analyzed: age, smoking, history, size of the defect, and administration of postoperative radiation therapy (Tables 1, 2, and 3). Comparative statistical analysis (*P* ≤ 0.05) between the variety of flaps was performed using a Students-*t*-test for two point discrimination, and light touch sensation, age, smoking, and length of followup.

Fisher's exact test analysis was used to evaluate hot-cold discrimination and the effect of using postoperative radiation therapy. Quality of life questionnaire was performed to assess articulation, oral continence, and aesthetic satisfaction in patients (in the appendix). Patients were evaluated at postoperative 3 and 6 months for the above mentioned tests. The relationship between cigarette smoking and sensory reinnervation has been evaluated and described in Table 2.

## 3. Results

Seventy-three noninnervated flaps showed comparable results with 52 innervated flaps for the lower lip reconstruction at the time of evaluation, a minimum of 6 months after the reconstruction. A statistical difference between innervated and noninnervated flaps was not seen.

Sensory recovery and sensation to touch was followed in order by two point discrimination and warm versus cold discrimination.

The earliest return of sensation to touch was recorded 3 months after surgery in patients with Nakajima fan flaps (Figures 3(a), 3(b), and 3(c)), and in 3 patients with Karapandzic fan flaps, the latest 24 months with radial forearm free noninnervated flap (Figures 4(a), 4(b), 5(a), 5(b), and 5(c)) postoperatively [5, 6].

Five patients failed to regain any flap sensation after followup of 25 and 18 months, respectively, especially in patients reconstructed with pectoralis major myocutaneous flaps and radial forearm free flaps.

68% of the patients showed some sensibility to touch in pectoralis major myocutaneous flaps eventually. Overall there was a strong trend for sensory recovery in fasciocutaneous flaps over myocutaneous flaps (depressor anguli oris flap (Figures 5(a), 5(b), and 5(c)), submental island flap, and pectoralis major myocutaneous flap) (p:0.09). The patients in our study did tend to perceive functional improvement when recipient site sensation was improved.

Two point discrimination tests showed sensibility of the transposed flaps comparable to the original cheek (cheek 2–12 mm), flap average of 10 mm (6–14 mm).

In patients who received radiotherapy, xerostomia was an additional factor complicating oral function since radiotherapy in the head and neck region causes saliva production problems (Table 4).

Touch sensation and temperature perception were significantly decreased when patients had received postoperative radiation therapy (*n* = 18).

**Figure 1:**
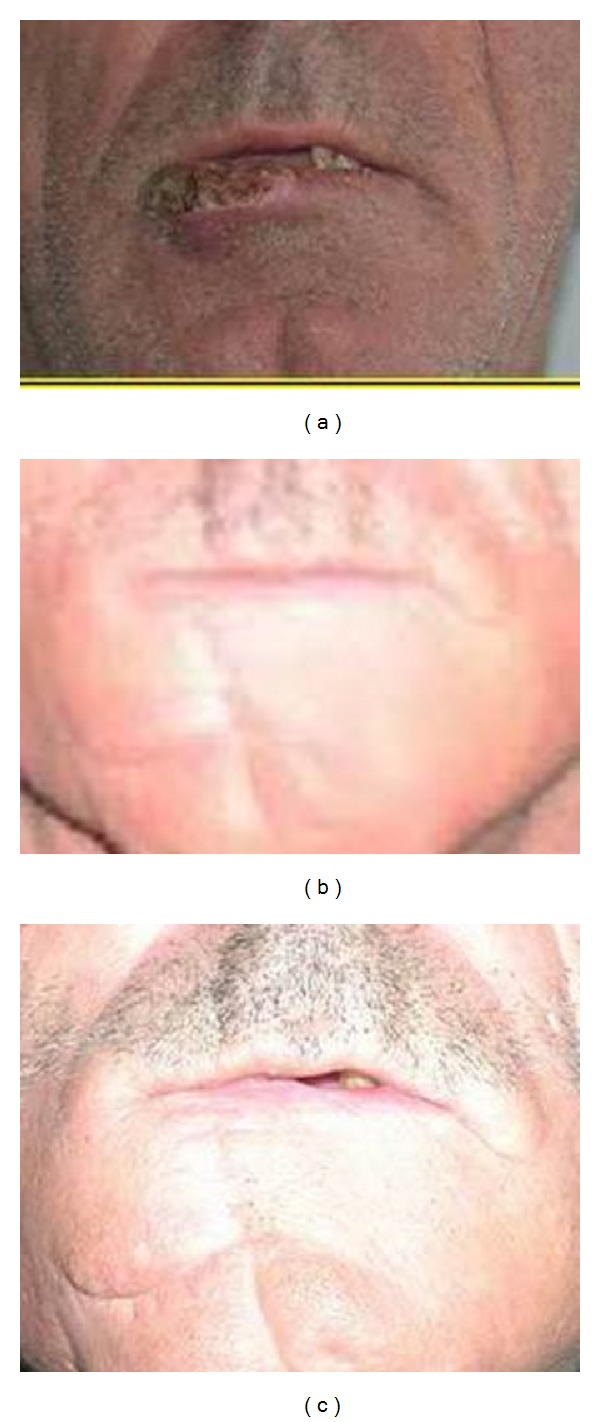
(a) Frontal view of patient with lower lip squamous cell carcinoma. (b) Postoperative view of unilateral depressor anguli oris flap. (c) Close-up view of unilateral depressor anguli oris flap.

**Figure 2:**
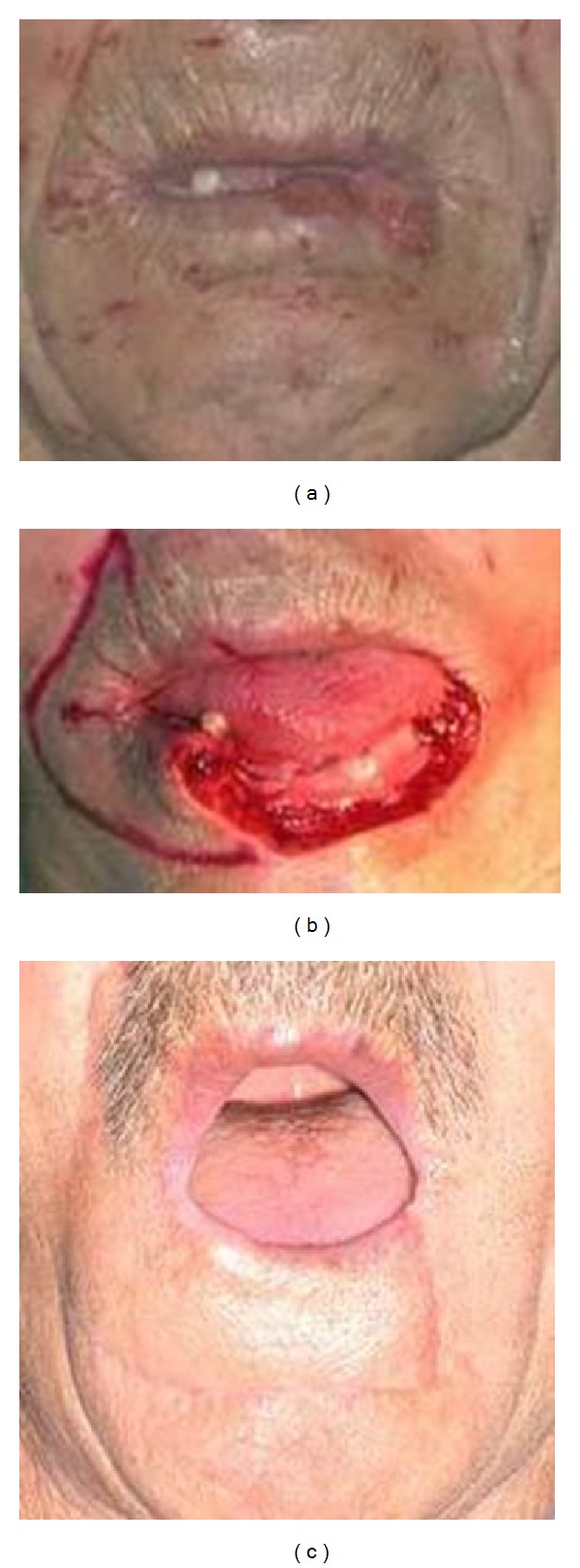
(a) Frontal view of patient with lower lip squamous cell carcinoma. (b) Peroperative view of unilateral fan flap. (c) Postoperative mouth-opening of the fan flap.

**Figure 3:**
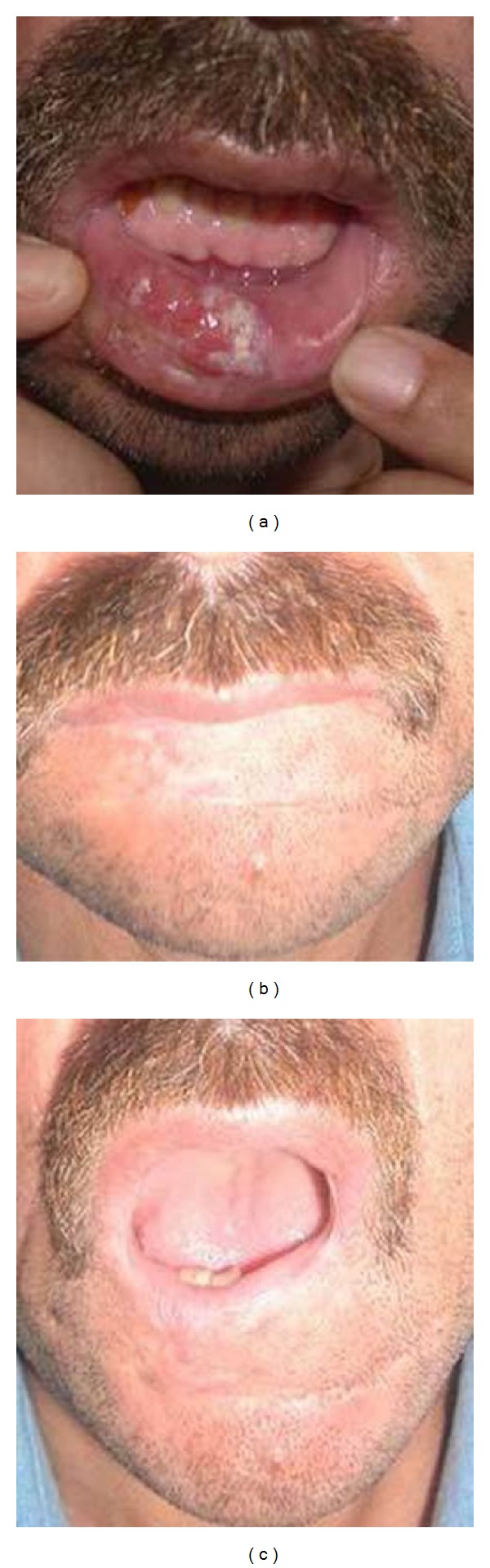
(a) Frontal view of patient with lower lip squamous cell carcinoma. (b) Postoperative view of bilateral karapandzic flap. (c) Postoperative mouth-opening of the karapandzic flap.

**Figure 4:**
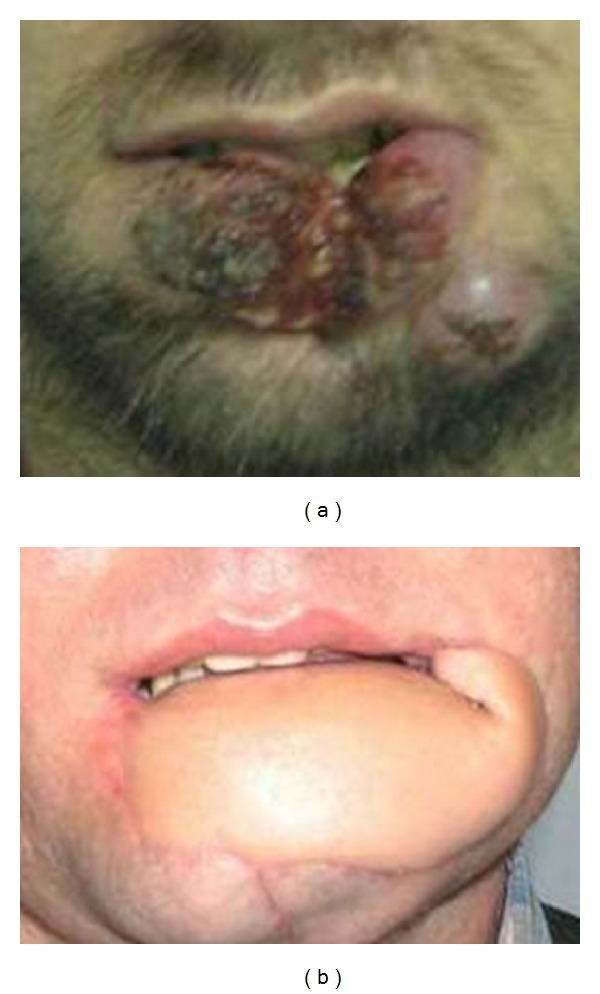
(a) Frontal view of patient with lower lip squamous cell carcinoma. (b) Postoperative view of radial forearm free noninnervated flap.

**Figure 5:**
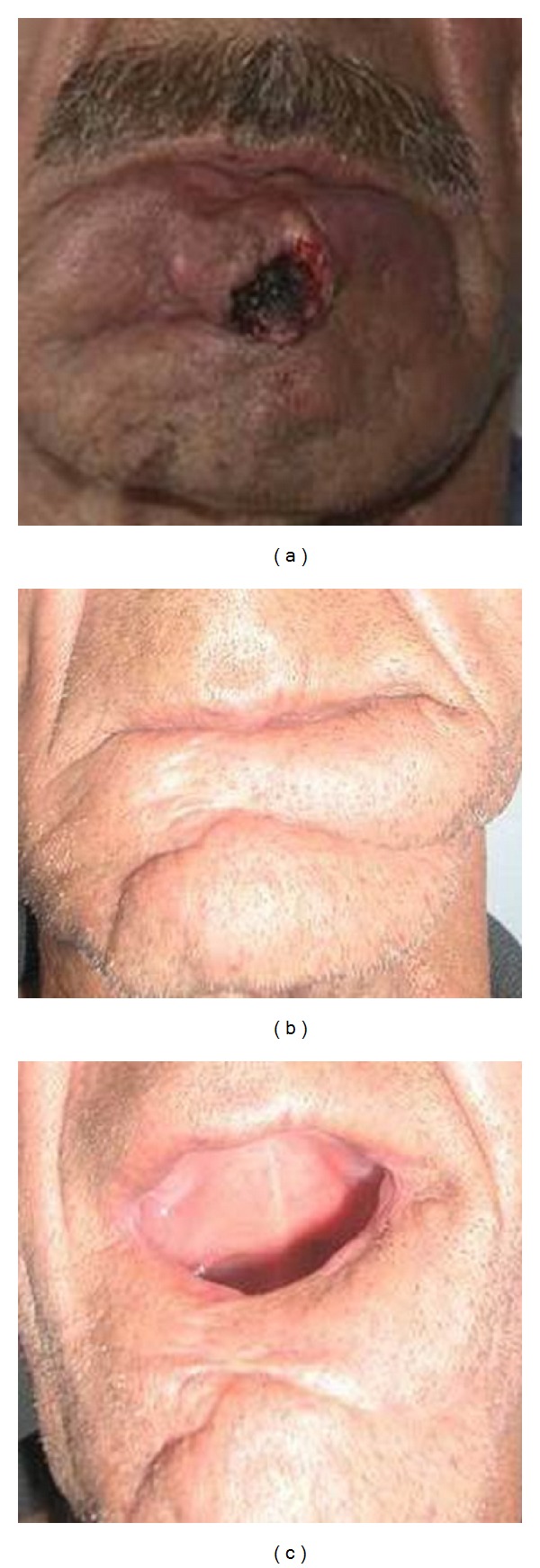
(a) Frontal view of patient with lower lip squamous cell carcinoma. (b) Postoperative frontal view of bilateral depressor anguli oris flap. (c) Postoperative mouth-opening in bilateral depressor anguli oris patient.

Patients had more satisfactory aesthetic results with fan flaps and depressor anguli oris flaps Figures 1(a), 1(b), 1(c), 2(a), 2(b), and 2(c).

We thought that patient age is closely related to postoperative oral function and sensory recovery of the flaps and we tried to demonstrate this relationship in our study.

Patients over the age of 58 showed relatively slower sensory recovery than others. Furthermore, flap thickness, quality of the recipient bed, and regional innervation are thought to play a role in the sensory recovery of noninnervated flaps. In our study we demonstrated this by the recovery rate of pectoralis major myocutaneous flap and submental island, and radial forearm flaps since they showed significantly slower recovery to superficial touch. There was a tendency for younger patients (*P* = 0.09) to have better articulation and oral continence postoperatively.

The results of our study in a fairly large group of patients revealed that there was not a significant sensory recovery in innervated flaps for lower lip reconstruction; these flaps included in this study being depressor anguli oris flaps, karapandzic flaps, nakajima and fujimori flaps, and submental island flaps.

## 4. Discussion

Nerve regrowth was investigated by several authors throughout the literature [7]. Shindo et al. (1955) investigated the differences in subjective sensibility of facial skin reconstruction versus oral cavity reconstruction with noninnervated skin flaps [8, 9].

Dellon (1988) proposed an association between eventual sensation and the number of sensory fibres connected with the receptor area [10]. The most important factor for natural recovery of noninnervated flaps appears to be the axonal sprouting from the recipient bed to the surface of the flap permitting axonal ingrowth [11].

Although complete recovery over the entire flap does not occur especially in free or pedicled flaps with a thick skin paddle, Hoppenjei et al. reported reinnervation in 5 patients reconstructed with pectoralis major myocutaneous flaps which showed some sensibility to touch [12].

The extent of trigeminal nerve branches that are resected with the tumor is also very important in axonal sprouting. The size of defects is important in sensory recovery. Shindo et al. reported that the degree of natural sensory recovery was greatest when flaps were used for smaller defects because spontaneous reinnervation depends on residual nerve population [7].

Histochemical studies of human skin grafts and flaps provide a basis for an understanding of the mechanism of sensory recovery in noninnervated flaps [13]. Dykes et al. obtained incisional biopsies from nine patients who had undergone skin grafting and found significant histochemical evidence of regenerating nerves at the bed and margins of the skin grafts 3 weeks after surgery [14, 15]. Chemotactic factors in the orientation of neural regeneration are suggested in this phenomenon. The phenomenon of reinnervation by the surrounding tissues has been described by Vriens and Close [16, 17].

The pattern of sensory recovery found in this series of patients of innervated and noninnervated flaps used for total lower lip reconstruction is quite different from others reported in the literature.

For example, the return of sensation to touch in our noninnervated flap patients was comparable and almost equivalent to innervated flaps. In our patients, sensation to touch was noted as soon as 3 months after surgery in patients with Nakajima and Karapandzic fan flaps and as late as 24 months postoperatively in radial forearm free flaps [18].

Similar to the findings in our study, improvement in two point discrimination in transferred flaps compared with that in the donor area has been reported. Cordeiro et al. assumed that this phenomenon was due to the wide cortical representation of these areas [19, 20].

The return of flap sensation in our study did correlate statistically (*P* = 0.045) with both articulation and oral continence. However, multiple factors affected the recovery of articulation and oral continence among which are effects of postoperative radiation therapy (pain, reduction of saliva), the resolution of postoperative and postradiotherapy edema, and the patient's ability to adapt to their new anatomy.

In our study, sensory recovery was more likely to occur after fan flaps (*n* = 86) than after myocutaneous flaps (*n* = 24). This relative lack of sensory recovery following myocutaneous flaps has been reported by Turkof et al. as well [5]. Turkof tested sensory recovery in 16 free myocutaneous flaps used for the lower extremity reconstruction. After followup of 18 months only 4 patients (25%) had recovery of touch and two point discrimination. Our findings support this observation.

## 5. Conclusion

This study compares sensory recovery after total lower lip reconstruction in a wide variety of flaps including bilateral depressor anguli oris flap, submental island flap, bilateral fan flaps, radial forearm flap, and pectoralis major myocutaneous flaps in a large number of patients.

We believe that our conclusions will help explain some of the differing reports in the literature on the sensory recovery of noninnervated flaps and will help the surgeons in their everyday practice when confronted with a total lower lip reconstruction.

## Figures and Tables

**Table 1:** Age and temperature perception on the lower lip with noninnervated flaps.

	Control	Fan flaps	Noninnervated radial forearm flap	Total
<60 yrs	20	10	7	37
>60 yrs	12	6	5	23

**Table 2:** Smoking and temperature perception on the lower lip with noninnervated flaps.

	Control	Fan flaps	Noninnervated radial forearm flaps	Total
NonSmokers	13	12	7	32
Smokers	19	4	5	28

**Table 3:** Radiation therapy and temperature perception on the lower lip with noninnervated flaps.

	Fan flaps	Noninnervated radial forearm flaps	Total
Nonirradiated	4	6	10
Radiated	1	4	5

**Table 4:** Incontinence frequency in noninnervated and innervated flaps.

	Depressor anguli oris	Karapandzic flap	Nakajima flap	Fujimori gate flap	Submental island flap	Fan flap	Pectoralis major flap	Radial forearm flap
Incontinence	2	4	1	1	1	12	3	8

## Appendix

Questionnaire

*Eating*

Do you have trouble with drinking?
To what extent have you changed your meals since the operation?
Do you have a dry mouth?
Do you splutter during speaking?
Do you have problems with drooling? On which side?

*Speech*

Do you have trouble speaking clearly?
Has your speech changed since the operation?
Can you make yourself comprehensible during a telephone-conversation?

*Aesthetics*

How would you rate your present appearance?
Do you find the scar(s) in your face annoying?
Does your appearance have a negative effect during your everyday-life?
